# Multidisciplinary Approach in Diagnosing Patients with Mental Health and Intellectual Disability

**DOI:** 10.1192/j.eurpsy.2022.178

**Published:** 2022-09-01

**Authors:** K. Krysta

**Affiliations:** Medical University of Silesia, Department Of Rehabilitation Psychiatry, Katowice, Poland

**Keywords:** intellectual disability, interdisciplinary support, mental health

## Abstract

It is possible to properly diagnose and treat a person with intellectual disability and mental health problems, but it requires a lot of work on the part of a doctor, nurse, professional medical care, proper rehabilitation and cooperation with a speech therapist, psychiatrist and many others. To improve the functional capacity of this person, it requires the help of a wide variety of professionals to meet their needs. One of the examples of interdisciplinary cooperation in this area is the “Circles of Support” program implemented by the Polish Association for People with Intellectual Disability in Poland. Support circles assume the creation of a group of supporters around a person with a disability, in accordance with their needs, combining formal (family doctor, psychiatrist, psychologist, personal assistant, social worker, therapist, lawyer) and informal support (family, friends, acquaintances, neighbors) , salesperson in your local store). The support is strictly focused on the needs of a person with a disability, and according to their individual preferences, it is fully inclusive. This goal can be achieved by preparing a person with a disability and the environment in a way that enables them to function safely in their local community based on the concept of supportive circles. The only way to build a system of care for of people with intellectual disabilities and their relatives is to rely on networks of connections - people and institutions, on their real commitment and on working out mechanisms supporting the empowerment of people with disabilities - in life, physical, financial matters.

**Disclosure:**

No significant relationships.

